# A Vulcanite Plate Retained in the Stomach

**Published:** 1871-12

**Authors:** 


*A Vulcanite Plate Retained in the Stomach*—Dr. Parker in the "Canada Medical Journal," relates the case of a lady, aged 25 years, of delicate constitution, who swallowed a set of three teeth on vulcanite, in March 1870, which is yet retained in the stomach. After the accident she experienced great suffering for a time, but was relieved by opiates and farinaceous food. Dr. Parker closes his account of the case as follows:

"The question arises—What will become of this foreign body if it is not passed 'per vias naturales?' And a second enquiry very naturally follows the first—What will become of the patient if it remains in the alimentary canal? If I am correctly informed, the material of which it is composed is not likely to be dissolved by the action of gastric juice, or by any of the secretions it may come in contact with, should it pass the pylorus. Dr. Cogswell in the article published in the "Canada Dental Journal," says:

"I felt desirous to know what mineral acids would dissolve vulcanite rubber, hence I experimented with the various muriatic, sulphuric, and nitric acids, found the two former had no effect upon the piece placed in it, but by applying nitric acid and chloroform, after 24 hours the piece had become quite like a sponge in softness, could easily express the coloring material from it, and in drying it could be rubbed up like powder between the fingers." If these strong mineral acids have failed to dissolve, or chemically change the material of which vulcanite is composed, I think we may safely conclude that the secretions of the digestive organs will hardly be able to accomplish it, and that the plate in question will, if not passed "per rectum," long continue in the canal without material alteration.

In reply to the second question—What will become of the woman should the foreign body continue in the canal? No certain statement can be given; but bearing in mind the history of recorded cases, somewhat analogous in their general features to that now under consideration, it may be remarked that it is possible, and even probable, that this vulcanite plate and teeth may be retained for years without destroying life, or even producing very alarming symptoms. On the other hand, grave symptoms may unexpectedly present themselves; the patient's life may be placed in jeopardy; or death may suddenly occur from inflammation, from ulceration, and perforation, or from its becoming impacted and obstructing the canal.

Dreading these not improbable contingencies, I objected to her being sent across the Atlantic to her friends in England, shortly after the accident occurred, on the ground that sea-sickness, if troublesome and violent, would be likely to produce irritation and perhaps fatal consequences.

The practical lessons to be learned from this case, are:

1st. That badly fitting plates holding artificial teeth are unsafe, and should not be worn—especially at night.

2nd. That much larger bodies than we would suppose, may find their way—accidentally or otherwise—into the stomach.

3rd. That when received there, even large and irregularly shaped bodies, may—and often do—remain for a length of time without producing alarming symptoms.

September 14th, 1871. I heard from this woman about the first of the present month, at which time she was a resident in the State of Rhode Island. She still wears the plate in the alimentary canal, and says that her health is quite as good as it was prior to the accident."

